# The global prevalence, formation, and evolutionary inference of bacteria co-carrying the *bla*_NDM_ and *mcr* resistance genes

**DOI:** 10.3389/fmicb.2025.1693785

**Published:** 2025-11-17

**Authors:** Boqian Wang, Kexin Li, Mingliang Chen, Jingjing Fu, Rui Zhang, Wanqiu Liu, Yuxin Wang, Zhixi Peng, Aimaiti Buaijier, Xinru Zhao, Hongbin Song, Hongguang Ren, Xiaofeng Hu

**Affiliations:** 1 Academy of Military Medical Sciences, Beijing, China; 2 Chinese PLA Center for Disease Control and Prevention, Beijing, China; 3 School of Public Health, China Medical University, Shenyang, China; 4 Institute of Pathology and Southwest Cancer Centre, Southwest Hospital, Army Medical University, Chongqing, China; 5 Department of Chemical Defense Medicine, School of Military Preventive Medicine, Army Medical University, Chongqing, China; 6 State Key Laboratory of Trauma, Burn and Combined Injury, Third Military Medical University, Chongqing, China; 7 Department of Epidemiology, School of Public Health, Southern Medicial University, Guangzhou, China; 8 School of Public Health, University of South China, Hengyang, China

**Keywords:** *bla*_NDM_, *mcr*, co-resistant bacteria, epidemiological analysis, genetic dynamics

## Abstract

**Introduction:**

The global dissemination of bacteria harboring *bla*_NDM_ and *mcr* genes, which confer co-resistance to carbapenem and polymyxin antibiotics, poses a catastrophic threat to public health due to the ineffectiveness of last-line antibiotics.

**Methods:**

This study integrated epidemiological and genetic analysis of 1,156 bacterial genomes from global databases and our *de novo* sequencing.

**Results:**

We demonstrate that the current “human-animal-environment” transmission drives their rapid geographical expansion and dynamic succession of dominant bacterial lineages (predominantly *Escherichia*, *Enterobacter*, and *Klebsiella*) over time. Notably, we identified several pieces of evolutionary evidence to elucidate the genetic dynamics of co-resistant bacterial formation as well as related plasmids and chromosome fusion. Additionally, we find that both broad-host and narrow-host plasmids are closely associated with these phenomena, but possess distinct genetic functions.

**Discussion:**

These findings elucidate the urgency of region-tailored surveillance, highlighting the need to target high-risk plasmid types and restrict non-therapeutic antibiotic use in agriculture to delay the “no-drug-available” crisis.

## 1 Introduction

Bacterial resistance has long been recognized internationally as a major threat to human health and public safety (Moellering, 2010; Walsh et al., 2011). Since the first discovery of *bla*_NDM_-carrying bacteria in 2008, their ability to hydrolyze carbapenem antibiotics has compromised the final therapeutic defense against gram-negative bacterial infections. In clinical practice, polymyxin antibiotics–despite their significant nephrotoxicity–have been employed as an emergency treatment for such antibiotic-resistant bacteria (Liu et al., 2016; Nang et al., 2021). However, the first identification of an *Escherichia coli* strain co-harboring *bla*_*NDM–9*_ and *mcr-1* in 2014, which confers resistance to both carbapenem and polymyxin antibiotics, has left clinicians with no viable therapeutic options (Yao et al., 2016). Of greater concern, bacteria harboring both *bla*_NDM_ and *mcr* genes (co-resistant bacteria) have been identified in Malaysia (Yan et al., 2021), Russia (Sulian et al., 2020), Vietnam (Le-Vo et al., 2019), South Africa (Mmatli et al., 2025) and other countries. These cases highlight the rapid spread of co-resistant bacteria across different environments and species, emphasizing the urgent need for global surveillance and intervention to prevent further dissemination of these dangerous pathogens.

Therefore, characterizing the current epidemiological status of these co-resistant bacteria represents an immediate research priority. Building upon this, systematic investigation of their emergence mechanisms is essential to elucidate whether they follow a unified evolutionary pattern, share ancestral lineages, or conversely, have evolved independently across different regions. Additionally, further analysis is warranted to determine whether *bla*_NDM_ and *mcr* genes are acquiring novel evolutionary adaptations within co-resistant bacteria that may potentiate their pathogenicity and dissemination efficiency. Addressing these challenges may clarify two key aspects: (1) the molecular mechanisms of cross-species transmission, and (2) pivotal evolutionary events driving antimicrobial resistance.

Current research mainly focuses on bacteria carrying either *bla*_NDM_ or *mcr* genes, preliminarily elucidating their origin and development individually (Acman et al., 2022; Wang et al., 2018). Related researches on co-resistant bacteria primarily focus on the isolation and characterization of individual clinical strains (Han et al., 2020; Lu et al., 2022; Mmatli et al., 2025; Sun et al., 2023; Tang et al., 2023). In these isolates, *bla*_NDM_ and *mcr* genes were typically located on distinct plasmids (Han et al., 2020; Sun et al., 2023; Tang et al., 2023), with chromosomal integration observed in a minority of cases (Lu et al., 2022; Mmatli et al., 2025). Furthermore, a hybrid plasmid co-harboring both *bla*_NDM_ and *mcr* genes was identified for the first time in a pet-origin *Escherichia coli* strain, and the mechanistic role of insertion sequences in plasmid fusion events was comprehensively investigated (Sun et al., 2016). Interestingly, some studies have found that strains from poultry sources are highly homologous to clinical isolates of co-resistant bacteria, and they can also be transmitted across species through the poultry industry chain, hospital environments, and migratory birds (Liu et al., 2024; Wang et al., 2017). Previous researches, constrained by limited sample sizes, failed to provide comprehensive insights into either the global epidemiology or molecular evolution of co-resistant bacteria. Critical knowledge gaps persist regarding: (i) the genomic drivers of co-resistance emergence, and (ii) the adaptive evolution of resistance genes in co-resistant strains.

In this study, we established a comprehensive genomic database of co-resistant bacteria based on NCBI and in-house isolated strains. We initiated our study with a systematic analysis of the global epidemiological profile of co-resistant bacterial strains. Subsequently, we expanded our dataset by retrieving all *bla*_NDM_- or *mcr*-harboring plasmids from the NCBI database. Through plasmid similarity identification, we successfully traced the evolutionary origins of some co-resistant bacterial strains. The results demonstrate that co-resistant bacteria did not evolve through a unified mechanism or shared ancestry, but rather emerged via independent regional evolutionary events. While these strains have caused localized outbreaks, fortunately, they have not yet established persistent dominant populations. Finally, we further traced evolutionary trajectories of resistance genes within certain co-resistant strains, identifying key genomic events including plasmid fusion and chromosomal integration. These intraspecies and interspecies transmission processes were predominantly mediated by specific mobile genetic elements, such as *IS26* and *IS3*. Our study concludes that co-resistant bacteria evolved independently, with resistance genes spread via plasmids and mobile genetic elements, without establishing persistent dominant populations.

## 2 Results

### 2.1 Global dataset of co-resistant bacteria

**FIGURE 1:**
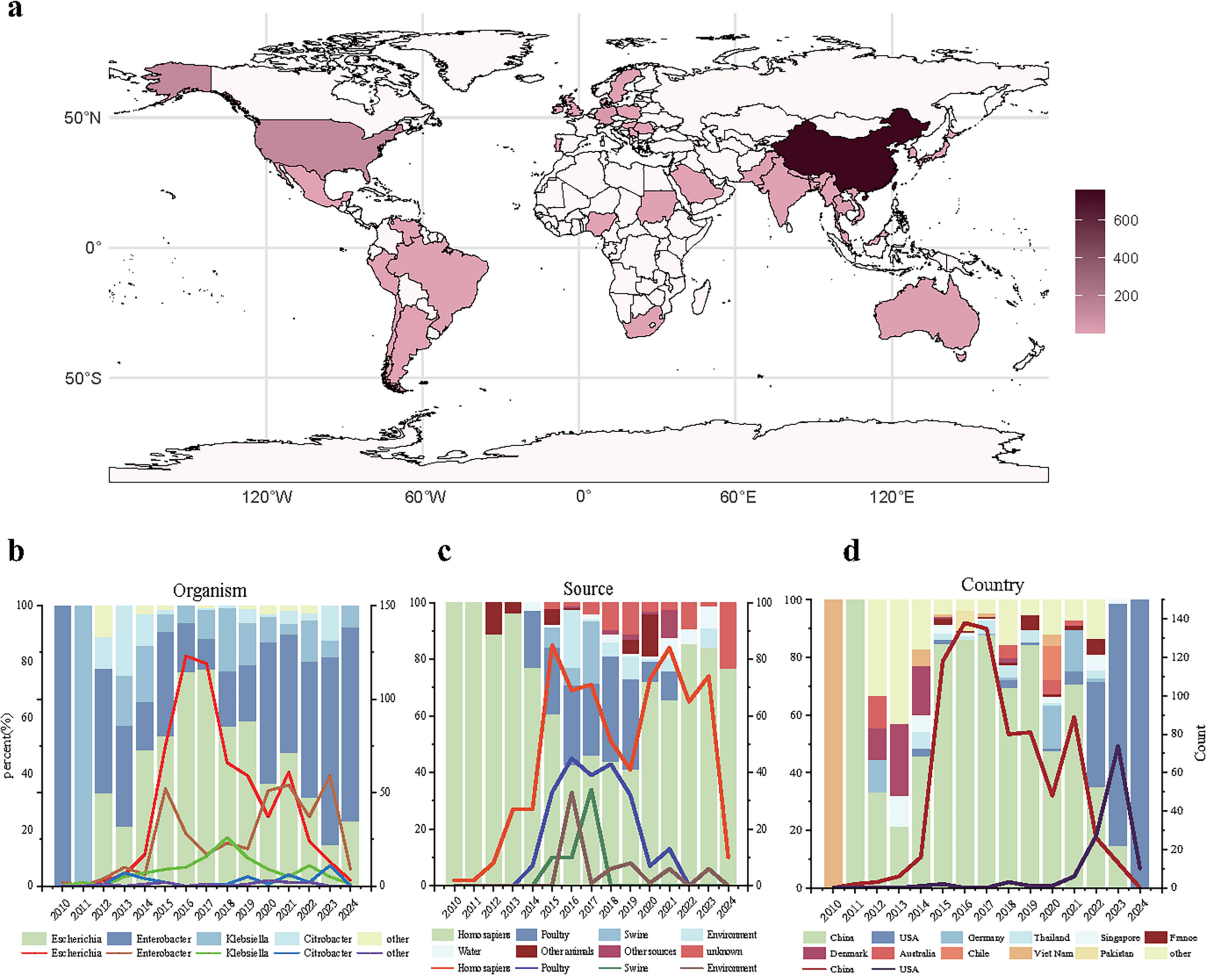
Basic epidemiological distribution of co-resistant bacteria. **(a)** Geographical distribution of co-resistant bacteria; **(b)** Distribution of co-resistant bacteria genera over time, where the *x*-axis represents the collection time, the left *y*-axis represents the proportion of each genus’ strains in each year, and the right *y*-axis represents the number of strains of the four main genera collected each year; **(c)** Collection sources of co-resistant bacteria over time, where the *x*-axis represents the collection time, the left *y*-axis represents the proportion of strains from different sources in each year, and the right *y*-axis represents the number of strains collected from the four main sources each year; **(d)** Collection numbers of co-resistant bacteria in different countries over time, where the *x*-axis represents the collection time, the left *y*-axis represents the proportion of strains collected from different countries each year, and the right *y*-axis represents the number of strains collected from the two countries with the highest collection numbers each year.

We compiled a dataset containing 1,156 bacterial strains, each of which carries at least one *bla*_NDM_ and *mcr* gene (Figure 1). The dataset includes 1,128 strains from NCBI Pathogen Detection and 27 strains from NCBI GenBank with assembled genomes published. Besides, we include a self-collected strain isolated from an inpatient in China and assembled *de novo* using SOAPdenovo. The dataset includes co-resistant bacteria from 39 countries across six continents, with the majority collected from Asia (*n* = 858) particularly from China (*n* = 732), the United States (*n* = 117), Germany (*n* = 37), and Thailand (*n* = 24) (Figure 1a). It covers 12 different bacterial genera, mainly *Escherichia* (*n* = 607), *Enterobacter* (*n* = 372), *Klebsiella* (*n* = 121), and *Citrobacter* (*n* = 41) (Figure 1b). In terms of strain origin, humans, birds, and pigs are the main host sources, with some strains also collected from the environment and water (Figure 1c). It indicates potential cross-host transmission among humans, animals and environment, leading to the spread of co-resistant bacteria. Finally, the highest number of strains appeared in 2016 (Figure 1d). Since the first discovery of co-resistant strains in 2010, the number gradually increased until 2016, followed by a fluctuating decline. This trend should be interpreted cautiously: it may reflect true epidemiological changes (e.g., reduced polymyxin use in agriculture) or sequencing artifacts (e.g., increased NGS accessibility after 2014, mandatory data submission in some countries). Distinguishing these factors requires long-term active surveillance, which is currently limited.

Initially, co-resistant bacteria carrying the *bla*_NDM_ and *mcr* genes were only found in Vietnam (Chavda et al., 2016) and China (Yao et al., 2016). Through epidemiological analysis, our study finds that nowadays co-resistant bacteria have a higher incidence in countries such as China, the United States, Germany, and Thailand, and are primarily found in the genera *Escherichia*, *Klebsiella*, and *Enterobacter*. From 2012 onward, *Escherichia* in China gradually became the main source of co-resistant bacteria, but during 2022–2023, *Enterobacter*-resistant bacteria in USA environmental samples caused a small-scale increase and eventually replaced *Escherichia* as the dominant genus. These strains are mainly transmitted through foodborne infections from birds, pigs, and humans, as well as environmental and waterborne transmission.

### 2.2 Distribution characteristics of plasmid types carrying *bla*_NDM_ and *mcr* genes in co-resistant bacteria

**FIGURE 2:**
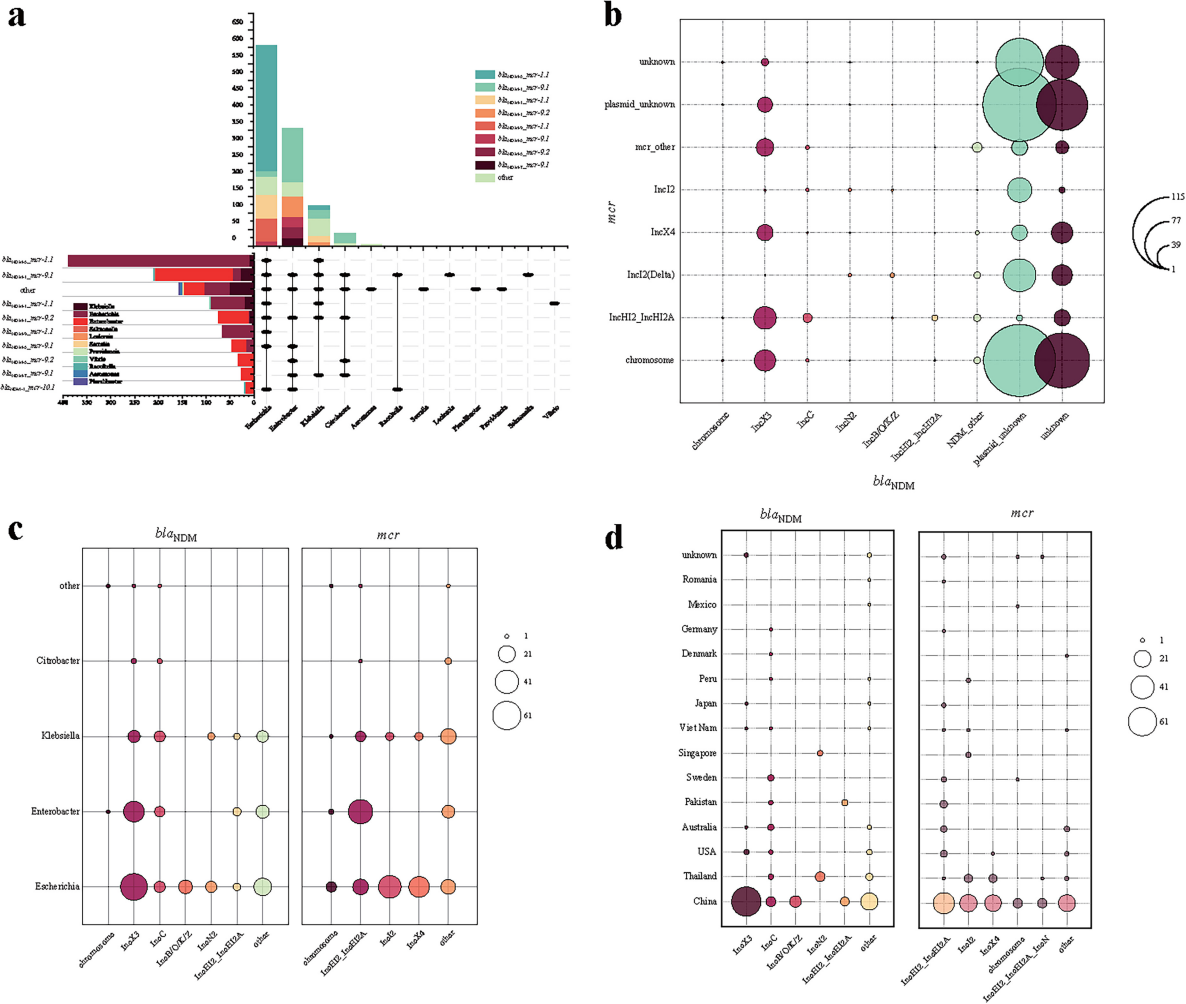
Description of plasmid characteristics carrying *bla*_NDM_ and *mcr* genes in co-resistant bacteria genera. **(a)** Combination and number distribution of *bla*_NDM_ and *mcr* genes in different bacterial genera of co-resistant bacteria; **(b)** Combination and number distribution of *bla*_NDM_ and *mcr* genes in chromosomal and plasmid types in different bacterial genera of co-resistant bacteria; **(c)** Number distribution of plasmid types carrying *bla*_NDM_ and *mcr* genes in different bacterial genera of co-resistant bacteria; **(d)** Number distribution of plasmid types carrying *bla*_NDM_ and *mcr* genes in different countries of co-resistant bacteria.

Among the 1,156 bacterial strains, the subtypes of both *bla*_NDM_ and *mcr* genes were precisely determined in 1,104 strains, which include 69 identified subtype combinations, with *bla*_*NDM–5*__*mcr-1.1* (*n* = 389) and *bla*_*NDM–1*__*mcr-9.1* (*n* = 238) being the most common (Figure 2a). The dominant combinations also vary among different bacterial genera: In *Escherichia* species, the dominant combination is *bla*_*NDM–5*__*mcr-1.1*, and the combination is mainly found in *Escherichia* species; In *Enterobacter* species, the dominant combination is *bla*_*NDM–1*__*mcr-9.1*.

Among the 1,104 bacterial strains above, the types of *bla*_NDM_- and *mcr*- harboring plasmids were precisely determined in 203 strains, and 13 strains hold *bla*_NDM_ or *mcr* gene in chromosome (totally 216 strains, Figure 2b). Regarding plasmids carrying only the *bla*_NDM_ gene, the plasmid types are primarily composed of IncX3, IncC, IncHI2, IncN2, IncB/O/K/Z, IncFIB, and IncFII, which is consistent with the current global trends (Wang et al., 2018). Regarding plasmids carrying the *mcr* gene, the plasmid types are mainly composed of IncI2, IncHI2, IncX4, IncN, IncFIA, IncFII, and IncP-1. Overall, 73 different combinations of replicons were identified (denoted as “*bla*_NDM_ plasmid type–*mcr* plasmid type”). The most common combination is IncX3-IncHI2 (35/203), with other important combinations including IncX3-IncX4 (25/203), IncC-IncHI2 (14/203), IncB/O/K/Z-IncI2 (11/203), and IncN2-IncI2 (10/203).

Some plasmids mentioned above are broad-host-range, such as IncC and IncP, which could facilitate the movement of resistance genes to other hosts, potentially expanding their transmission range. The dominate plasmid types differ by bacterial genera (Figure 2c) and countries (Figure 2d). For example, from the perspective of genera, *bla*_NDM_-harboring IncB/O/K/Z plasmids are only identified in *Escherichia*, and *mcr*-harboring IncI2/IncX4 plasmids in *Escherichia* and *Klebsiella* (Figure 2c). From the perspective of countries, *bla*_NDM_-harboring IncC plasmids are identified in most countries, while IncN2 plasmids are only found in Thailand and Singapore (Figure 2d).

Additionally, there are plasmids with multiple replicons, such as the co-presence of IncFIB and IncFII, or IncFIB and IncHI1B, which significantly enhance the range and capability of resistance gene transfer (Lin et al., 2025). Notably, there are also instances where both genes are located on the same plasmid (*n* = 12), which is either with multiple replicons (*n* = 3) or IncHI2 (*n* = 9), facilitating their horizontal transmission, or on the chromosome (*n* = 1), potentially allowing for their vertical transfer (Mei et al., 2022). Such variability implies a complex and dynamic evolutionary trajectory in co-resistant bacterial populations.

### 2.3 The formation process of co-resistant bacteria harboring *bla*_NDM_ and *mcr* genes

**FIGURE 3:**
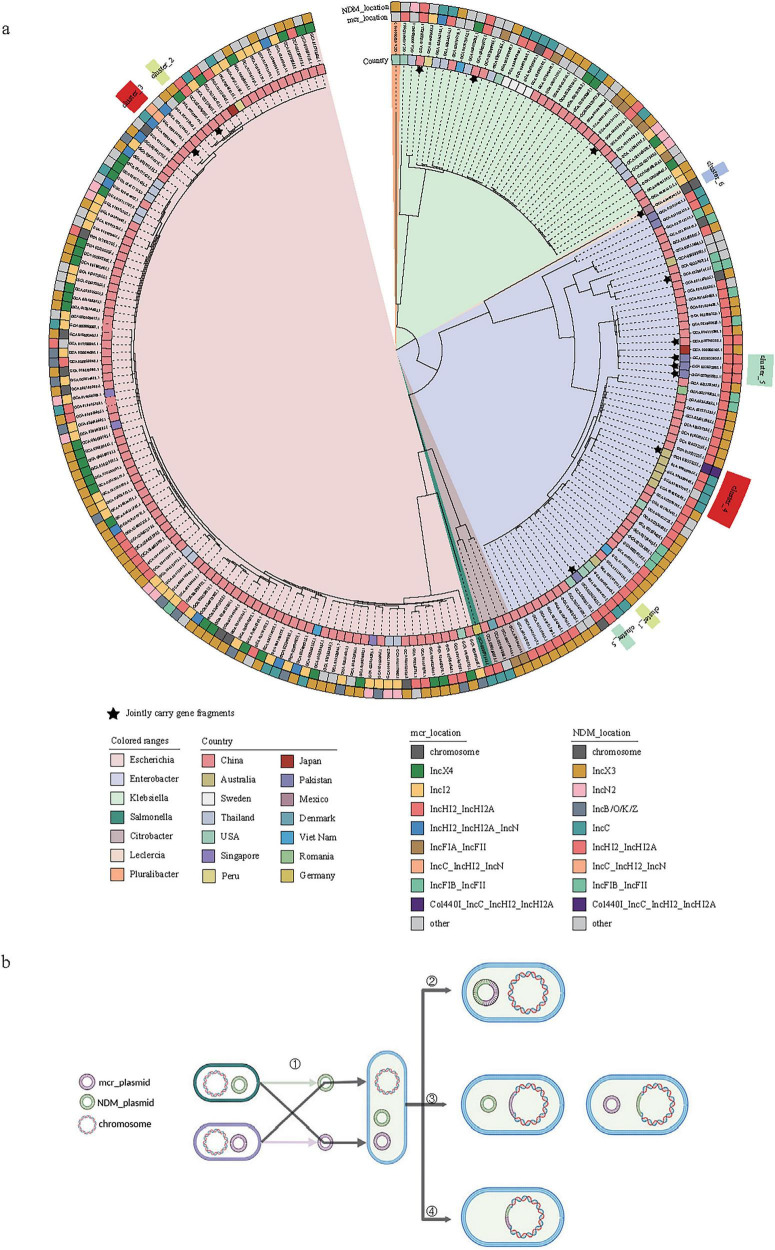
Phylogenetic analysis and gene transfer mechanism of co-resistant bacteria carrying *bla*_NDM_ and *mcr* genes. **(a)** Core genome phylogenetic tree of 216 strains of co-resistant bacteria carrying *bla*_NDM_ and *mcr* genes with distinguishable plasmid types, where the outer circle represents the plasmid types of *mcr* and *bla*_NDM_ genes; **(b)** Sources of *bla*_NDM_ and *mcr* genes in plasmids and potential changes after transfer to the same strain in co-resistant bacteria, categorized into the formation of co-resistant bacteria, subsequent transfer of *bla*_NDM_ and *mcr* genes to the same plasmid, transfer of *bla*_NDM_ or *mcr* genes to the chromosome, and simultaneous transfer of *bla*_NDM_ and *mcr* genes to the chromosome. The star symbol indicates that the *bla*_NDM_ and *mcr* genes are located in the same plasmid.

To investigate the evolutionary process of co-resistant bacteria, we firstly constructed a phylogenetic tree for the 216 strains based on bac120 (Figure 3a). Bac120 (Chaumeil et al., 2022) refers to a set of single-copy marker genes that are widely present in bacterial genomes and used for phylogenetic analysis and classification of bacterial genomes. These genes are highly conserved and exist as a single copy in the bacterial domain, making them suitable for constructing phylogenetic trees and species classification. Among these 216 strains, we observed that some co-resistant strains carrying either *bla*_NDM_ or *mcr* harboring plasmids individually shared high similarity with plasmids from strains carrying only *bla*_NDM_ or *mcr* genes, providing evidence for the formation of co-resistant bacteria (Figure 3b). Following co-resistance emergence, core genome phylogenetic analysis revealed that at the terminal branches, the vast majority of strains carried *bla*_NDM_ or *mcr* positive plasmids of distinct types, with only a minority sharing identical plasmid types. Comparative analysis of highly similar plasmids identified processes including plasmid fusion and chromosomal integration events (Figure 3b).

**FIGURE 4:**
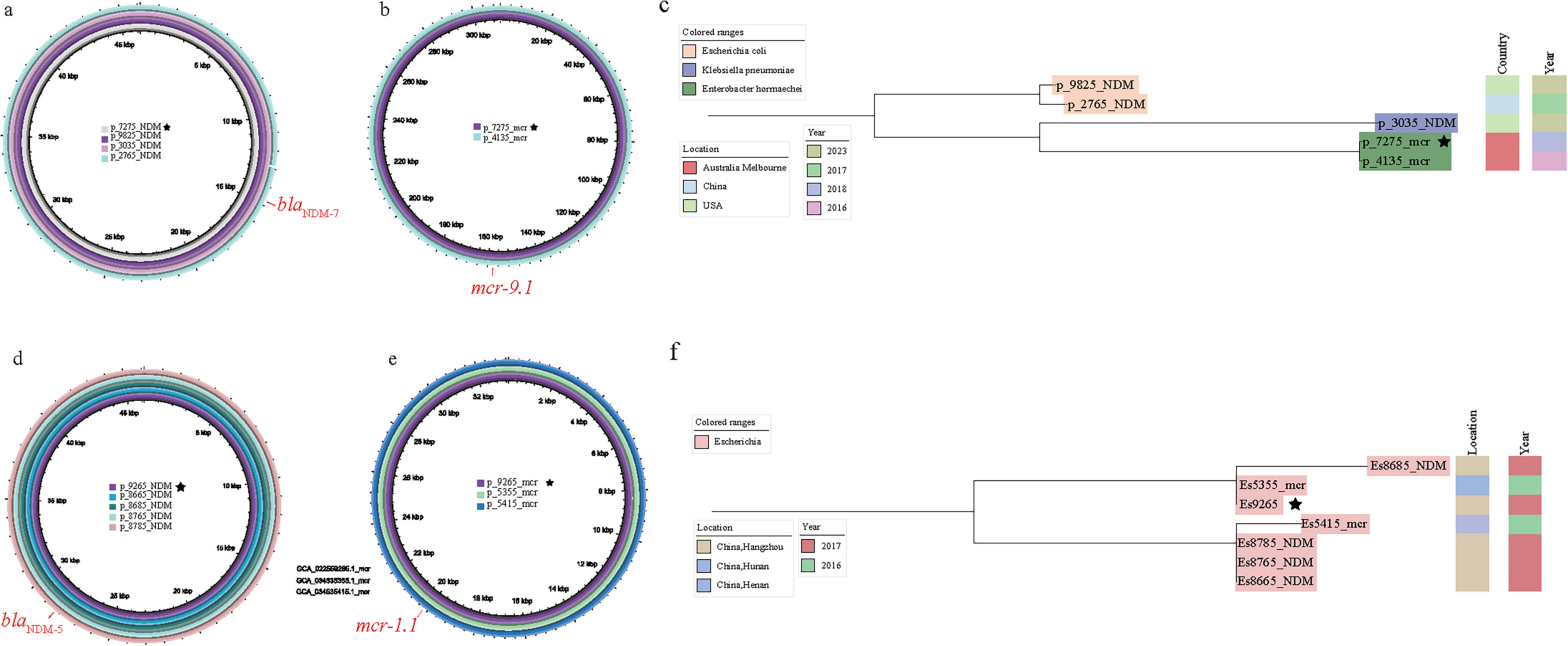
Analysis of the association between the plasmid carrying mode of *bla*_NDM_ and *mcr* genes and the evolution of the core genome (the star symbol indicates that the *bla*_NDM_ and *bla*_NDM_ genes located in the same strains). **(a)** The results of carrying the *bla*_NDM_ plasmid p_En7275_NDM; **(b)** The results of carrying the *mcr* gene plasmid p_ En7275_mcr; **(c)** The results of comparing En7275 with the other four core genomes; **(d)** The results of carrying the *bla*_NDM_ plasmid p_Es9295_NDM; **(e)** The results for the plasmid carrying the *mcr* gene p_Es9295_mcr; **(f)** The results of comparing Es9295 with the other four core genomes search results.

To elucidate the evolutionary events leading to bacterial co-resistance formation, we used Skani to compare *bla*_NDM_- or *mcr*-harboring plasmids in co-resistant bacteria with the plasmid databases carrying either *bla*_NDM_ or *mcr* genes. For co-resistance strain En7275, on the one hand, its *mcr*-carrying plasmid p_En7275_*mcr* can be traced back to an *Enterobacter* (En4135, GCA_016774135.1) strain collected in Melbourne, Australia, in 2016 (p_En4135_mcr). On the other hand, its *bla*_NDM_-carrying plasmid p_En7275_NDM perfectly matched the plasmids in two *Escherichia* and one *Klebsiella* strains distributed in the United States and China (Figures 4a, b). Comparatively, En7275 showed greater similarity to the core genome of En4135, indicating a horizontal transmission of *bla*_NDM_-carrying plasmid and a vertical transmission of *mcr*-carrying plasmid in this case (Figure 4c).

Besides, for the co-resistance strain Es9295 collected from Hangzhou, China, its *bla*_NDM_-carrying plasmid p_Es9295_NDM perfectly matched the *bla*_NDM_-carrying plasmids in four bacteria strains GCA_022558765.1(Es8765), GCA_022558785.1(Es8785), GCA_022558685.1(Es8685), GCA_022558665.1 (Es8665) were collected from the same time and hospital as Es9295. Meanwhile, its *mcr*-carrying plasmid p_Es9295_*mcr* perfectly matched the plasmids found in strains from Henan, China (GCA_034535415.1, Es5415) and Hunan, China (GCA_034535355.1, Es5355). Although they were not found in the same region, Es9295 and Es5415 had more closer core genomes, suggesting that this strain may have acquired the *bla*_NDM_-carrying plasmid after initially harboring the *mcr*-carrying plasmid (Figures 4d, e, f).

### 2.4 The evolutionary process of *mcr*-carrying and *bla*_NDM_-carrying plasmids in co-resistant bacteria

**FIGURE 5:**
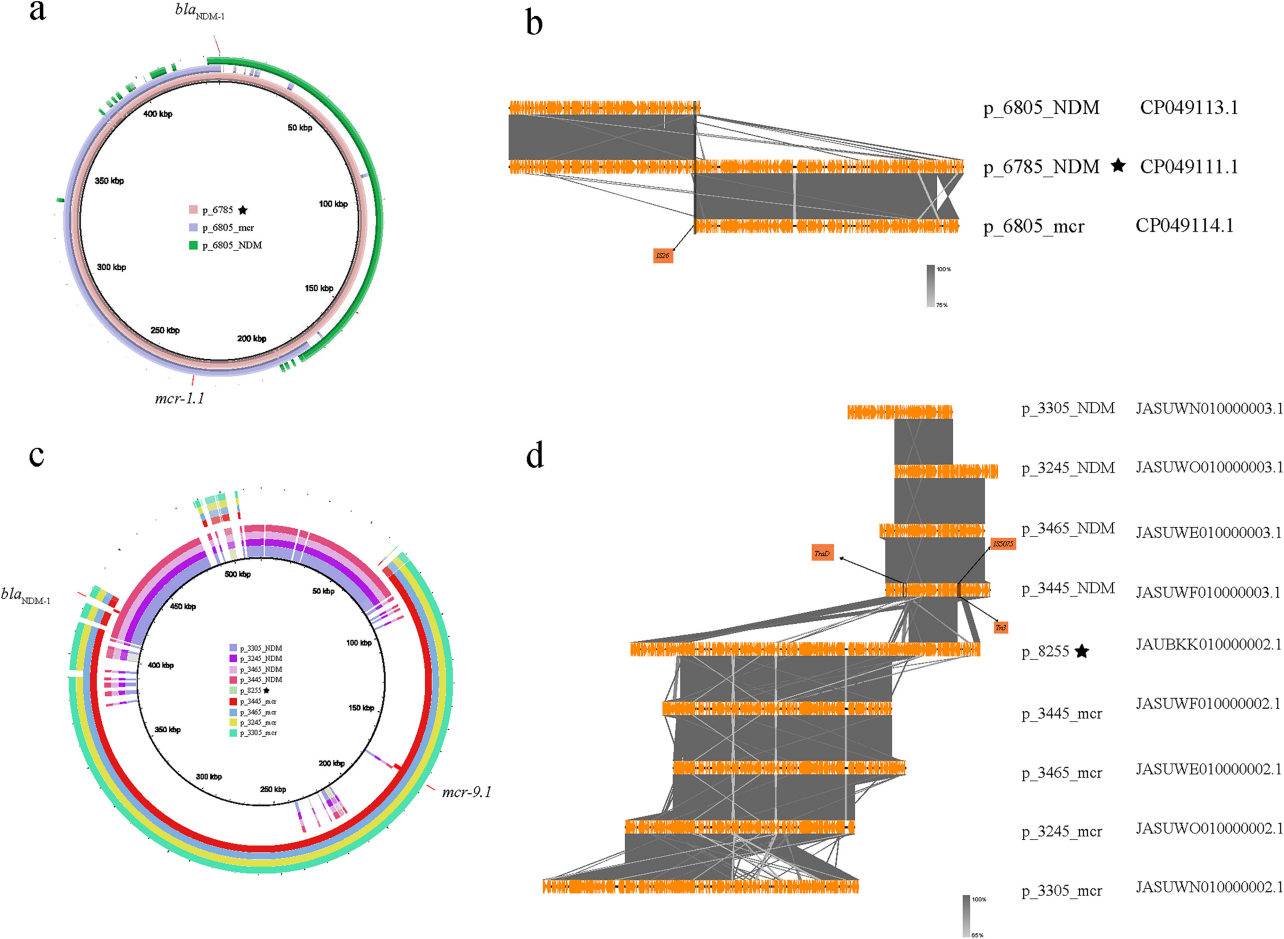
Homology comparison and structural differences between *bla*_NDM_ and *mcr* genes in co-existing plasmids and single-carrying plasmids (the star symbol indicates that the *bla*_NDM_ and *mcr* genes are located on the same plasmid). **(a)** The homology between p_Es6785 and p_6805_NDM and p_6805_mcr; **(b)** The alignment of the differences between p_Es6785 and p_6805_NDM and p_6805_mcr; **(c)** The plasmids carrying both *bla*_NDM_ and *mcr* genes in En8255 and those carrying only *bla*_NDM_ and *mcr* genes in En3305, En3465, En3445, En3245; **(d)** The alignment of the differences between p_8255 and those carrying only *bla*_NDM_ and *mcr* genes in En3305, En3465, En3445, En3245.

Plasmids carrying the *bla*_NDM_ and *mcr* genes in co-resistant bacteria undergo subsequent evolution. In the phylogenetic tree constructed based on bac120 from the 216 strains mentioned above (Figure 3a), we found that at the terminal branches of closely related core genome evolution, some strains exhibit plasmids fusion generating plasmid carrying both the *bla*_NDM_ and *mcr* genes (Figure 3b). For instance, two strains Es6785 and Es6805 from China could explain the fusion process of plasmids. Except for a few recombination events, p_Es6805_NDM (IncC with *bla*_*NDM–1*_) and p_Es6805_mcr [IncHI2(A) and IncN with *mcr-1.1*] can be combined through *IS26*-mediated recombination and form p_Es6785 (Figures 5a, b).

A similar situation is observed in five sequences from Australia, where the *bla*_NDM_ and *mcr* genes in En3305, En3465, En3445, and En3245 are located on two different plasmids (Figures 5c, d). These plasmids carrying the *bla*_NDM_ or *mcr* genes separately have long regions of high similarity. In contrast, in En8255, the *bla*_NDM_ and *mcr* gene are both located on the p_En8255. When comparing these plasmids, we found that two breaks occurred at a specific point on the *bla*_NDM_-carrying plasmid, and a *mcr*-carrying plasmid inserted at this site. Specifically, the first break point in p_En3445_NDM (the *bla*_NDM_-carrying plasmid in GCA_030283445.1) corresponds to a gene sequence encoding a coupling protein (*TraD*) involved in the conjugative transfer system, which helps form the conjugation complex to transfer plasmid DNA from the donor to the recipient bacterium (Schröder et al., 2002). An insertion of p_En3445_mcr (the *mcr*-carrying plasmid in GCA_030283445.1) occurs between the two break points. A subsequent break point shows the presence of insertion sequences *IS5075* and *Tn3*, with an unknown foreign sequence inserted between the two break points. The combined effect of these two break points led to the formation of *bla*_NDM_ and *mcr* co-carrying plasmid p_En8255.

**FIGURE 6:**
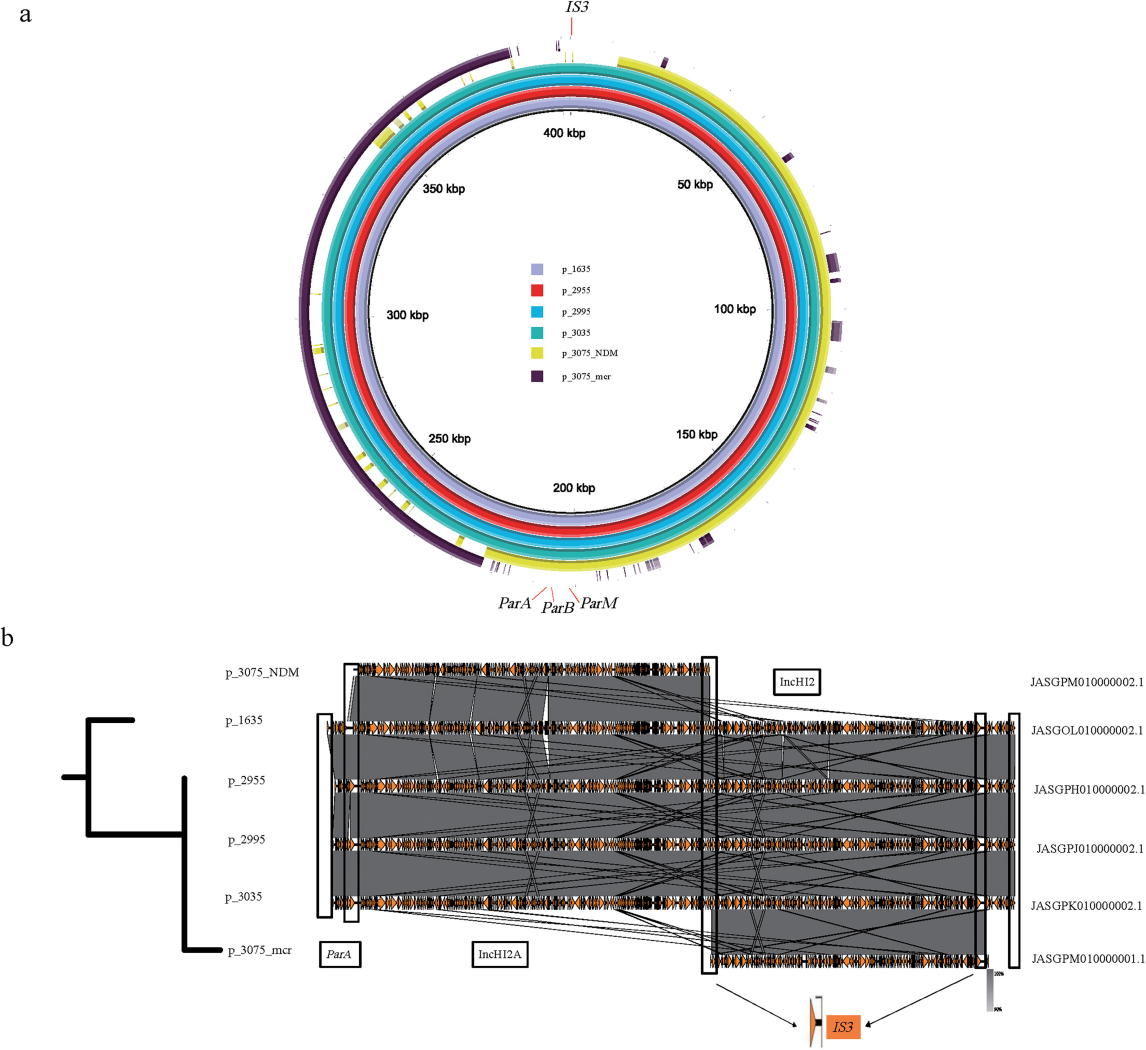
Comparison of the genomic structure and fusion analysis of plasmids carrying *bla*_NDM_ and *mcr* genes. **(a)** En3075, which carries the *bla*_NDM_ and *mcr* gene fragments separately, partially matches with En1635, En2955, En2995, and En3035, which also carry the *bla*_NDM_ and *mcr* gene plasmids. Among them, p_En1635, p_En2955, p_En2995, and p_En3035 carry both the *bla*_NDM_ and *mcr* gene plasmids, while p_En3075_mcr and p_En3075_NDM carry the *mcr* gene and *bla*_NDM_ fragments, respectively. **(b)** The left part is a comparison of the core genome tree of En3075 with En1635, En2955, En2995, and En3035. The right part is a comparison of gene fragments, in which about 4% of the front part of p_En3075_mcr has a high homology with the above-mentioned four plasmids, and p_En3075_NDM also has a high homology with the above-mentioned four plasmids. After fusion mediated by *IS3*, the two parts have more than 80% homology with the above-mentioned four plasmids. The difference lies in the lack of the *IS3*-mediated region (front end, about 5%) and the *ParA* protein-mediated region (tail end, about 5%).

In addition to plasmids fusion, resistance gene can also be transferred from plasmids to chromosomes. The fragment carrying the *mcr* gene in En3075 was recognized as a chromosome c_En3075_mcr with a length of 4970668 bp, while the fragment carrying the *bla*_NDM_ was recognized as a plasmid p_En3075_NDM with a length of 210482 bp. Moreover, in En1635, En2955, En2995, and En3035, both the *bla*_NDM_ and *mcr* genes are located on the same plasmid, and the sequence similarity between these plasmids is very high (Figure 6a). Through the bac120 phylogenetic analysis of the above five strains of bacteria, we found that En3075 had a higher degree of evolution of the core genome compared to the other four strains (as reflected in the evolutionary tree on the left side of Figure 6a). Furthermore, through BLAST analysis, we found that the head part of c_En3075_mcr and the p_En3075_NDM had similar matches with the above four similar plasmids, although there were some sequence differences (Figure 6b). These differences mainly occurred in certain specific areas, and in these difference areas, we found the existence of *IS3* transposable elements. *IS3* elements may be associated with the breakage of plasmids, becoming a potential factor causing plasmid breakage. Moreover, the tail regions of four similar plasmids all contain the *ParA* protein. This *ParA* protein interacts with the DNA binding protein *ParB* and a specific DNA sequence *ParS* to form an allocation system, which participates in the process of plasmid distribution in cells. Therefore, in the bacterial replication process, under the joint action of the above two situations, the drug-resistant genes are transferred between the plasmid and the chromosome.

## 3 Discussion

The interconnected transmission of co-resistant bacteria carrying *bla*_NDM_ and *mcr* genes across human, animal, and environmental interfaces has spurred dynamic shifts in their geographic distribution and dominant bacterial genera over time. While our study provides insights into the dissemination characteristics of these co-resistant bacteria at a global scale, we acknowledge limitations due to uneven sequencing capacity and data submission practices—for example, regions with robust AMR surveillance (China, US, Germany) are overrepresented, while data from low- and middle-income countries remain scarce. Thus, our observations do not fully capture the complete global dissemination pattern but highlight key trends supported by available data. Initially confined to specific strains like *Enterobacter* in Vietnam and *Klebsiella* in China, these pathogens now circulate globally, with *Escherichia*, *Klebsiella*, and *Enterobacter* emerging as primary genera (Figures 1a, b). Regions such as China, the USA, Germany, and Thailand exhibit distinct patterns: China’s high prevalence of these co-resistant bacteria in *Escherichia* is linked to the extensive use of specific antibiotics in poultry and pig farming—particularly polymyxins (the selective pressure for *mcr* genes, which confer polymyxin resistance) and carbapenems (the selective pressure for *bla*_NDM_ genes, which confer carbapenem resistance)—as well as subsequent environmental contamination via livestock waste, while the USA indicates *Enterobacter* dominance driven by hospital-associated and localized environmental transmission (Figures 1c, d; Klein et al., 2024). These changes reflect evolving selective pressures from antibiotic usage, agricultural practices, and public health policies, highlighting the One Health continuum’s role in shaping resistance epidemiology (Himanshu Prudencio et al., 2022).

Notably, our dataset contains a disproportionately high proportion of isolates from China (≈63%, 732/1,156), which may introduce bias into conclusions regarding global distribution. This imbalance likely reflects regional differences in sequencing capacity (e.g., widespread application of NGS in Chinese AMR surveillance), data submission incentives (national AMR programs mandating public data sharing) and research focus. While our key findings (e.g., plasmid-mediated transmission, human-animal-environment spread) align with reports from other regions (e.g., *Escherichia* carrying *bla*_NDM–5_/*mcr-1.1* in Southeast Asia), the generalizability to areas with limited sequencing data (e.g., parts of Africa, South America) should be cautious. Future global surveillance needs equitable data collection to refine true global prevalence.

The most prevalent genetic combinations of *bla*_NDM_ and *mcr* genes include *bla*_*NDM–5*__*mcr-1.1* (predominant in *Escherichia*) and *bla*_*NDM–1*__*mcr-9.1* (common in *Enterobacter*), conferring robust resistance due to synergistic effects (Figure 2a). A longitudinal study on plasmids can enhance our understanding of the transmission and evolutionary patterns of the *bla*_NDM_ and *mcr* genes. Their widespread persistence is facilitated by plasmids such as IncX3, IncHI2_IncHI2A, and IncI2 (Figure 2b). Narrow-host-range plasmids (e.g., IncB/O/K/Z in *Escherichia*) dominate regional transmissions, adapting to specific environmental niches, whereas rare broad-host-range plasmids (e.g., IncX3, IncHI2_IncHI2A) enable cross-species spread, driving resistance across genera (Figures 2c, d). For example, IncX3 prevails in Chinese *bla*_NDM_ carriers, while IncHI2_IncHI2A dominates in the USA, Australia, and Pakistan for *mcr* transmission. In some strains, *bla*_NDM_ and *mcr* are located on the same plasmid, mainly the IncHI2 plasmid. This is partly due to the high genetic diversity of the *Enterobacteriaceae* family and the broad length range of these plasmids, which makes them key components in the formation of fusion plasmids (Algarni et al., 2024; Li et al., 2025). Although genomic data suggest that IncHI2-mediated co-localization may enhance the co-transmission of two resistance genes (through a single binding event), this hypothesis lacks the support of phenotypic data. Currently, although there are experiments quantifying the binding efficiency of strains with genes on different plasmids and the results show that they are not affected, the actual transmission remains unknown. This plasmid diversity, combined with clonal amplification and horizontal gene transfer, enhances multidrug resistance in interrelated ecosystems, while the functional role of co-localization of the same plasmid still awaits experimental verification (Sun et al., 2016; Wang et al., 2023). This plasmid diversity, combined with clonal expansion and horizontal gene transfer, amplifies multidrug resistance across interconnected ecosystems. Specific plasmids confer selective advantages to dominant clonal lineages in different regions, leading to temporal and spatial heterogeneity in their plasmid profiles (Stoesser et al., 2020), with local outbreaks further amplifying the spread of already dominant plasmids (Yang et al., 2013).

Introducing another plasmid may lead to plasmid loss or rapid adaptation, such as acquiring resistance and other accessory elements, and also provides opportunities for resistance to spread through transposition or recombination (Castañeda-Barba et al., 2024). In this way, new resistance genes can be established in another plasmid ecological niche. However, it is worth noting that plasmids in co-resistant bacteria undergo rapid dynamic changes during their adaptation to the environment and transmission across hosts, influenced by external environmental factors such as antibiotic pressure (Ma et al., 2023). Meanwhile, these changes are also constrained by the development and varying standards of sequencing technologies and assembly processes (Yang et al., 2025). The significant temporal and spatial differences of co-resistant bacteria strains (Figure 3a) make it difficult to determine uniform transfer patterns of *bla*_NDM_- and *mcr*-carrying plasmids. By comparing plasmids carrying *bla*_NDM_ and *mcr* genes with the core genome, we found that the trend of co-evolution does not apply to the overall group (Figure 3a), but only applies to certain branches of the evolutionary tree. Based on this, we searched for and analyzed similar plasmids carrying *bla*_NDM_ and *mcr* genes in co-resistant bacteria at the individual level. In En7275, p_En7275_mcr is highly similar to p_Es4135_mcr in En4134 (Figure 4b), which was discovered in Australia two years ago and is closer to the core genome than strains carrying *bla*_NDM_ alone (Figure 4c). This suggests that *Enterobacter hormaechei* with the IncHI2_IncHI2A plasmid carrying the *mcr* gene then acquired the IncX3 plasmid carrying *bla*_NDM_ which were found in China and the USA (Figure 4a). In Es9265, p_Es9265_NDM has four similar plasmids in the same hospital, which collected date was closer (Figure 4d), instead, similar plasmids of p_Es9265_mcr were found in Hunan and Hainan (Figure 4e), the core genome similarity was higher than strains carrying *bla*_NDM_ alone (Figure 4f). This strain of bacteria shows that after entering the hospital, the bacteria carrying *mcr* gene were influenced by the prevalence of bacteria carrying *bla*_NDM_ in the hospital, and the plasmids carrying *bla*_NDM_ were transferred to them, ultimately forming co-resistant bacteria. Although both IncX3 and IncHI2 are narrow host plasmids as mentioned above, they are “all star” drug-resistant plasmids that can shuttle between various genera of bacteria (Guo et al., 2022; Yang et al., 2022).

These strains spread via bird/pig-related foodborne routes and environmental/waterborne pathways. Our “poultry–human–environment” route inference is supported by bac120-based core genome alignment: human-derived Es9295 (Hangzhou) shows high bac120 similarity with poultry-associated Es5415 (Henan), and environment-derived En7275 (Australia) is closely related to poultry-derived En4135 (Australia) via bac120, with both pairs sharing homologous resistance plasmids (Figure 4). We acknowledge the need for further epidemiological data (e.g., source-tracing surveys) to validate this route.

The spread of resistance genes occurs not only through plasmids but also through other mobile structures like transposons and insertion elements (Rana et al., 2024). When a plasmid is introduced and adapted, plasmid fusion mediated by insertion sequences may occur. For example, a study reported the fusion of plasmids carrying *bla*_NDM_ and bla_*IMP*–4_ genes mediated by the IS26 element (Fang et al., 2024). The coexistence of *bla*_NDM_ and *mcr*-1 genes has been documented in bacterial isolates from various sources, highlighting the potential for these resistance genes to spread through plasmid-mediated mechanisms (Toribio-Celestino et al., 2024). This indicates that insertion sequences can play a significant role in the formation of multidrug-resistant bacteria by facilitating the recombination between different plasmids, thereby enhancing the resistance of the host bacteria. One study found that an IncX3 plasmid carrying *bla*_*NDM–5*_ and an IncX4 plasmid carrying *mcr*-1 in *Escherichia coli* merged through hybridization, integrating together via IS26 and the nic site of oriT to form an IncX3-X4 hybrid plasmid. This hybrid plasmid was also stably retained in the original *Escherichia coli* strain (Sun et al., 2016). In this study, plasmid fusion events were observed in Es6785/Es8255 (Figure 5), where smaller plasmids integrated through insertion sequences like IS26 and recombined into larger plasmids, allowing both the *bla*_NDM_ and *mcr* genes to coexist on the same plasmid. In addition to transposition, gene excision and gene module rearrangement mediated by homologous recombination between IS26 scattered on plasmids or genomes also drive bacterial evolution and there are experiments to prove it (Partridge et al., 2018; Zhao et al., 2021). Insertion events from plasmids to the genome can also occur, and En3075 may have undergone an IS3-mediated insertion event from plasmids to the chromosome (Figure 6). Studies suggest that when resistance genes are present on the chromosome, their transfer and expression may be more strictly regulated, indicating that gene transfer on the chromosome is strongly restricted by phylogenetic barriers (Ellabaan et al., 2021). Although plasmids carrying *bla*_NDM_ and *mcr* genes can undergo changes as described above, whether these changes affect the expression of resistance still requires further experimental verification.

In conclusion, this study provides an epidemiological analysis of bacteria co-carrying *bla*_NDM_ and *mcr* genes, along with a description of their plasmid characteristics, formation, and subsequent evolutionary development. Our findings reveal that the current “human-animal-environment” transmission have led to their rapid geographical expansion and continuous shifts in dominant bacterial lineages over time. It is noteworthy that we reconstructed multiple typical cases, providing a comprehensive depiction of the entire evolutionary trajectory—from the initial formation of co-resistant strains to subsequent genetic refinement—where plasmids and insertion sequences played a significant role (Figure 4b). Although co-resistant bacteria pose a significant threat, they are currently mostly sporadic, and there is no unified evolutionary law or a common ancestor, possibly due to the fitness cost of *bla*_NDM_ [accumulating toxic intermediates (López et al., 2019) or through an unknown mechanism of efflux pumps (Yoon et al., 2016)] and the fitness cost of *mcr* [mainly manifested in its impact on bacterial growth, virulence, and plasmid persistence (Yang et al., 2021)]. Nevertheless, it is crucial to take appropriate measures to control its spread. Existing research on *mcr* suggests that reducing the use of polymyxins can reverse corresponding antibiotic resistance (Guo et al., 2024). In addition to giving sufficient attention to the use of carbapenems and polymyxins as last-line antibiotics to combat infections, attention should also be paid to key plasmids (such as the prevalence of IncHI2 in regions with high non-therapeutic antibiotic use in agriculture).

## 4 Materials and methods

### 4.1 Comprehensive dataset construction and basic information statistics

First, we analyzed the bacterial genomic sequences previously collected from our sentinel hospital using BLAST (v2.12.0). The reference sequences used were *bla*_*NDM*–1_ and *mcr-1.1*, with the standard set as identity > 80% and coverage > 80%. We found a strain that simultaneously carried the *bla*_NDM_ and *mcr-9.1* genes. This drug-resistant bacterium was derived from a sputum sample of a female patient from Hainan in 2015. The genomic DNA was extracted from cultured bacteria using the QIAamp DNA Mini Kit (Qiagen, Inc., Valencia, CA, USA), and sequencing was performed on the Illumina HiSeq 2500 platform (Illumina, Inc., San Diego, CA, USA) by Novogene Co., Ltd. (Beijing, China), with an insert fragment size of 350 bp. *De novo* assembly was performed using the SOAPdenovo genome assembler (v2.04) (Li et al., 2010) with an average coverage of 110×. Scaffold construction and gap filling were carried out using SSPA (Boetzer et al., 2011) and GapFiller (Boetzer et al., 2011; Krawczyk et al., 2018), respectively. The plasmids pNDM-BTR, p112298-KPC, and pF8475 were used as references. Gap closure was performed using reference-guided assembly and manual inspection by remapping the raw reads to the plasmid sequences (Yang et al., 2018). The sequence was included in this study (see Supplementary File 1: 1_self_1).

Second, we conducted a comprehensive search in the NCBI Pathogen Detection database^1^ using the query: AMR_genotypes: *mcr* * AND AMR_genotypes: *bla*_NDM_. This search identified bacterial genomic sequences carrying both the *bla*_NDM_ and *mcr* genes, and a total of 1,128 related genome data were downloaded.

Finally, to reduce the omissions in NCBI Pathogen Detection due to the absence of annotated *bla*_NDM_ and *mcr* gene sequences, based on the NCBI refseq database, we performed a genomic sequence search using all known subtypes of the *bla*_NDM_ and *mcr* genes^2^ as references. The search criteria were set to identity > 80% and coverage > 80%. This search covered 46 bacterial genera. A total of 80,222 genomic sequences were downloaded from the NCBI database (as of 4 April 2024). Using all known subtypes of the *bla*_NDM_ and *mcr* genes as references, we performed a sequence search with the same criteria (identity > 80% and coverage > 80%) and identified 355 genome sequences that met the criteria. Of these, 328 sequences were already downloaded from Pathogen Detection, and 27 sequences were added to our dataset.

We integrated the genomic sequences obtained from the three parts above, removed duplicate downloads, and finally collected 1,154 unique genomic sequences. Using Python (code: 1_read_gbff.py), we extracted basic information from the annotation files (.*gbff* files), including collection time, country/province, source, genus, etc. This information was summarized (see Supplementary File 1: 2_Comprehensive dataset_1156) and visualized using R packages like ggplot2, rnaturalearth, and Origin.

### 4.2 Identification of plasmid types carrying *bla*_NDM_ and *mcr* genes

In order to accurately identify the subtypes of drug-resistant genes, blast analysis was performed on antibiotic-resistant strains carrying *bla*_NDM_ and *mcr* genes to screen for strains whose sequences completely matched the reference sequences of the two genes mentioned above. Using all drug-resistant gene subtypes as a database for matching, so it is reasonable to set the standard as identity = 100% and coverage = 100%, and these sequences were included in the analysis dataset (see Supplementary File 2: 1_ Analyzed_dataset_1104) and record the name of the gene fragment.

Afterward, we used Python (code: 2_process_fna_file.py) to batch extract the scaffolds where the *bla*_NDM_ or *mcr* genes are located, and some of the locations of the *bla*_NDM_ or *mcr* genes were marked as chromosome fragments (the results are detailed in Supplementary File 2: 2_chromosome); then we used Plasflow (vision:1.1.0) (Krawczyk et al., 2018) (a tool that uses machine learning methods to predict plasmid sequences from short-read assembly data) to infer the origin of the overlapping groups (the results are detailed in Supplementary File 2: 3_plasflow_1104), and according to the results, we screened the sequences located in the plasmid, and then used Plasmidfinder (vision:2.1.6) (Carattoli and Hasman, 2020) to identify the replicons with identity > 95%, and screened out those with at least one replicon, and determined the genotype of the fragment (the results are detailed in Supplementary File 2: 4_plasmidfinder_216) After screening, the distribution of *bla*_NDM_ and *mcr* simultaneously located in chromosomes or the same plasmids is shown in Supplementary File 2: 5_co-carrying_contig.

### 4.3 Description of plasmid distribution characteristics of *bla*_NDM_ and *mcr* gene-carrying strains

In order to clearly demonstrate the evolutionary relationship and characteristic distribution of co-resistant bacteria, and further determine the outbreak points or aggregation points, we systematically analyzed 216 strains in the Supplementary File 2: 4_plasflow_216. First, we used GTDB-Tk (version 2.4.0) (Chaumeil et al., 2022), with default parameters, to classify and construct a phylogenetic tree based on their bac120 marker genes. The bac120 gene is widely present in bacteria, and constructing a phylogenetic tree based on Bac120 can encompass many different bacterial groups within the bacterial domain, while maintaining a certain degree of sequence conservation. This ensures effective comparison across different bacterial species. At the same time, there are sufficient variations in the gene, which accumulate as bacteria evolve, recording genetic changes during the evolutionary process. This moderate conservation and variation make the Bac120 gene an ideal molecular marker for building phylogenetic trees, accurately reflecting the phylogenetic relationships between bacteria.

We then used the iTOL (Letunic and Bork, 2024) tool to visualize the constructed phylogenetic tree. We added multiple layers of annotation to the tree, including the genus distribution of co-resistant strains, the countries of origin, and the specific locations of the *bla*_NDM_ and *mcr* genes on the chromosome, to visually display the evolutionary relationships between different strains and the distribution characteristics of the resistance genes.

### 4.4 Plasmid source search for *bla*_NDM_ and *mcr* gene-carrying strains

To find the source plasmids of the *bla*_NDM_-and *mcr*-carrying plasmids in co-resistance strains, we performed separate searches using the queries: AMR_genotypes: *mcr* *; AMR_genotypes: *bla*_NDM_ to retrieve bacterial strains that individually carry the *bla*_NDM_ and *mcr* genes in the NCBI database Pathogen Detection. After removing the previously identified co-resistant strains, we downloaded the corresponding sequences. Among them, 24,663 genome sequences carried only the *bla*_NDM_ gene (see Supplementary File 3: 1_NDM_24663), and 13,206 genome sequences carried only the *mcr* gene (see Supplementary File 3: 2_mcr_13206). We then extracted the genomic fragments containing the *bla*_NDM_ and *mcr* genes.

Next, we used skani (version: 0.2.2) (Shaw and Yu, 2023) for alignment. skani has a notable advantage in query speed, and in addition to outputting ANI values, it also provides the alignment fraction to better assess the true similarity between the two genomes. It is also suitable for incomplete genomes. The ANI, Align fraction ref, and Align fraction query parameters were set to 100, and we filtered genome sequences that met these criteria. The basic information of the filtered sequences was extracted (this was achieved using code: 3_skani_and_search_gbff.py). In addition to plasmid similarity, we also extracted year and geographical information and compared it with the information of co-resistant bacteria. The geographical information may not be in the same location, which can be explained by the large flow of people, but the huge gap in years cannot be explained and will be discarded.

### 4.5 Fusion of *bla*_NDM_ and *mcr* gene-carrying plasmids and inference

There is a case of plasmid fusion carrying *bla*_NDM_ and *mcr* genes at the end of the evolutionary tree branch, and we want to determine its fusion process and structural changes, we used BRIG (Alikhan et al., 2011) to determine whether similar fragments exist, with the e-value set to 10e-5 to ensure that the identified similar fragments had high credibility, with other parameters set to default. We then performed a BLAST search on the selected sequences using escyfig (Sullivan et al., 2011), with the following parameters: min. length set to 500, max. e-value set to 0.0001, and min. Identity value set to 99 to ensure that the sequences of interest had extremely high similarity. The remaining parameters were set to default. Afterward, we selected the key fragments for annotation and labeling.

## Data Availability

The accession numbers of all 1,156 bacterial genomes obtained from the NCBI In review database are included in Supplementary File 2: 1_ Analyzed_dataset_1104. The *de novo* sequence in this study is available via https://ngdc.cncb.ac.cn/gwh/Assembly/64098/show. All software used in this research are described in the “4 Materials and methods” section, which are available from the GitHub website (https://github.com/likex903/1_NDM_mcr).

## Footnotes

1
https://www.ncbi.nlm.nih.gov/pathogens/

2
https://www.ncbi.nlm.nih.gov/refseq/
